# Phase Shift Cavity Ring-Down (PS-CRD) Absorption of Esters in the Near-Infrared and Visible Regions: Agricultural Detection and Environmental Implications

**DOI:** 10.3390/s25113448

**Published:** 2025-05-30

**Authors:** David Camejo, Carlos E. Manzanares

**Affiliations:** 1 Department of Chemistry and Biochemistry, Baylor University, 101 Bagby Avenue, Baylor Sciences Building, Waco, TX 76706, USA; david.camejo@lonestar.edu; 2 Department of Chemistry, Lone Star College-CyFair, 9191 Barker Cypress Road, Cypress, TX 77433, USA

**Keywords:** ester absorption, cavity ring-down, vibrational overtone, field detection, atmospheric reactions

## Abstract

**Highlights:**

**What are the main findings?**

- Optical pathlengths of 25 km are achieved with our PS-CRD technique.
- Absorption coefficients as low as 10^−10^ cm^−1^ are measured.

**What is the implication of the main finding?**

- Vibrational overtones of organic esters are recorded.
- Emissions of esters in the field can be detected and identified by the CRD technique.

**Abstract:**

A detailed description of the components of the CRD technique is presented and applied to the detection of organic esters. These molecules typically have a pleasant smell resembling the aroma of flowers and fruits and are responsible for many distinct odors in plants. They are emitted into the atmosphere by natural sources and human production. The weak absorption spectrum of the fifth vibrational overtone of ethyl, ethyl trimethyl, and tert-butyl acetate are recorded to show the sensitivity of the CRD technique. A description of a compact instrument to be used in the near-IR and visible regions will be presented for measurements of ester detection in the field. Potential chemical reactions of esters induced by visible light absorption in the atmosphere are discussed.

## 1. Introduction

### 1.1. Organic Esters

Esters whose functional group is (R-CO-OR) typically have a pleasant, fruity smell, often resembling the aroma of flowers and fruits, as they are commonly found in nature. In fruits, such as apples, pears, and bananas, esters are the primary constituents of their distinctive aroma. The alarm pheromones emitted by honeybees contain butyl acetate. These compounds serve a dual purpose in mature fruit: they attract animals and provide protection against pathogens [1]. In fruits like strawberries, they contribute to the overall blend of volatile compounds that create the scent [2]. Most volatile esters are characterized by fruity flavors [3]. Additionally, these compounds are not exclusive to fruits; they are also present in floral fragrances [4]. Vegetative parts of plants frequently emit esters, either continuously or in response to stress or insect attacks [5]. Thus, esters play a significant role in the interactions between plants and their surroundings. Esters are frequently used in artificial flavorings and fragrances to mimic these scents. In industry, ethyl acetate is a widely used solvent, especially for paints, varnishes, lacquers, cleaning mixtures, and perfumes. Tert-butyl acetate is also a solvent used in the production of lacquers, enamels, inks, adhesives, thinners, and industrial cleaners.

### 1.2. Background on Cavity Ring-Down Spectroscopy

**Figure 1:**
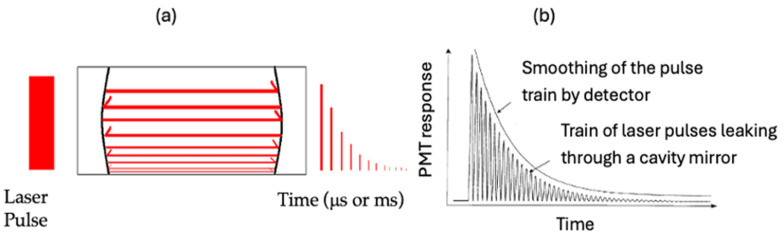
(**a**) Pulsed cavity ring-down diagram. (**b**) Exponential decay of laser light within the ring-down cavity.

Cavity ring-down (CRD) spectroscopy is a direct absorption method that can utilize either pulsed or continuous light sources. This technique offers significantly enhanced sensitivity compared to traditional absorption spectroscopy. The technique produces optical pathlengths that can extend over many kilometers within a compact physical area. The predominant detection techniques employed in CRD spectroscopy include the exponential method [6,7] and the phase shift method (PS-CRD) [8,9,10,11]. Typically, CRD spectroscopy comprises a laser source, an optical cavity, and a detector that measures the light exiting the optical cavity [12,13]. In our laboratory, we have used the exponential and phase shift methods. We illustrate the background of CRD following the dissertation of Perez-Delgado [14]. In this research, we show the latest improvements in design, particularly for the permanently aligned CRD cell and the PS-CRD technique. Figure 1a illustrates a typical pulsed cavity ring-down diagram. In a standard ring-down experiment, laser light is introduced into a cavity created by two high-reflectivity mirrors (over 99.99%). When the mirrors are aligned in a parallel arrangement, the incoming beam reflects repeatedly between them, the beam becomes trapped inside the cavity for a certain amount of time causing the long optical path length. Due to the finite reflectivity of the mirrors, a small amount of light leaks out of the cavity. Then, the photo-sensitive detector placed behind the output mirror records the intensity of the small amount of light transmitted through it. The light intensity is reduced by a given percentage on each round trip. The detector sees an exponential decay of light intensity. If short laser pulses are used, a very fast detector will see a train of pulses within an exponential decay envelope, but the time response of detection electronics usually means the pulses are smoothed into a single exponential curve. This is shown in Figure 1b

The intensity of the light transmitted (I) out of the cavity decreases as a function of time (t) and can be characterized as:

(1) $$I\left(t\right)=I_{0}e^{-(t/\tau )}$$

where *I*_0_ is the initial light intensity detected and τ is the decay constant. This decay constant (τ) is commonly referred to as the ring-down time, whose value can range from 1 μs to 100 μs or more, depending on mirror reflectivity. The exponential CRD detection method uses an exponential fitting of the output signal from the cavity to measure the decay constant.

**Figure 2:**
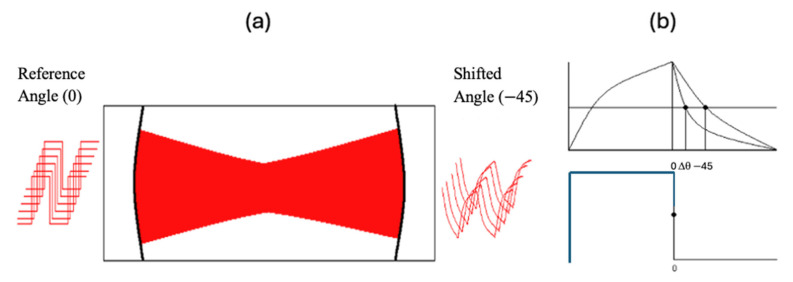
(**a**) Phase shift cavity ring-down diagram. (**b**) PS-CRD signal (**top**), reference signal (**bottom**).

PS-CRD measures the phase shift in a modulated laser beam from the output beam of the cavity with respect to the modulated beam, or reference beam, before entering the cavity. The phase shift ($\phi \left(\nu \right)$) is a function of the laser wavenumber (ν) and is related to the time that the light spends in the cavity or ring-down time (τ) by the equation: $tan\phi \left(\nu \right)=2\pi f\tau$, where *f* is the modulation frequency. In Figure 2a the modulated laser source is incident upon the optical cavity.

The output signal from the cavity in Figure 2a resembles a shark fin. This can be described as light building up in the cavity on the positive slope side and light leaking out of the cavity on the negative slope side. The phase shift angle is monitored on the negative slope of the exiting wave. A better picture of this signal is shown in Figure 2b. This figure illustrates the original signal (or reference signal) going into the cavity (bottom) and the phase shift signal detected after the beam traverses the cavity (top).

The absorption of light by a sample inside the cavity is described by the Beer–Lambert law, which gives a quantitative relationship between the intensity of a spectral feature and the frequency-dependent absorption properties of the sample:

(2) $$I=I_{0}e^{-(\epsilon cl)}$$

where (*I*) is the output intensity equal to the incident intensity (*I*_0_), the extinction coefficient of the sample (cm^−1^ M^−1^) at the wavenumber (ν) of the measurement (ϵ(ν)), the concentration of the absorber (c) and the sample pathlength (l). The product (ϵc) is the absorption coefficient ($\alpha \left(\nu \right)$) and the absorbance (A) is defined as (A = ϵcl). From this equation, if the pathlength is long, very small concentrations of the sample or very weak absorption cross-sections can be detected. Hence, CRD provides a long pathlength to make direct absorption measurements of either trace amounts or weak absorptions.

For a cavity filled with an absorbing gas, the ring-down time is [12,13]:

(3) $$\tau \left(\nu \right)=\frac{l}{c\left[\left(1-R\right)+\alpha \left(\nu \right)l\right]}$$

The time (τ) is related to the reflectivity of the mirrors (*R*), the speed of light (*c*), the absorption coefficient ($\alpha \left(\nu \right)$) of the sample as a function of the frequency (ν), and the length (l) of the optical cavity. The absorption spectrum of the background or empty cavity ${\left(c\tau_{0}(\nu )\right)}^{-1}=\frac{\left(1-R\right)}{l}$ is subtracted from the absorption spectrum of the sample plus the background ${\left(c\tau (\nu )\right)}^{-1}$ to obtain the absorption coefficient of the sample.

(4) $$\alpha \left(\nu \right)=\frac{1}{c\tau \left(\nu \right)}-\frac{1}{c\tau_{o}\left(\nu \right)}=\frac{\omega }{c}\left[\frac{1}{tan\phi \left(\nu \right)}-\frac{1}{tan\phi_{o}(\nu )}\right]$$

The first part of Equation (2) is for exponential CRD, the second part is for phase shift CRD. The calculated absorption coefficient ($\alpha \left(\nu \right)$) in cm^−1^ units is plotted as a function of the wavenumber (cm^−1^) of the laser to show the spectrum of the sample.

## 2. Materials and Methods

### 2.1. Materials

Ethyl acetate liquid (CAS No. 141-78-6, 99.9%, HPLC grade), ethyl trimethyl acetate (CAS No. 3938-95-2, 99%), and tert-butyl acetate (CAS No. 540-88-5, ≥99.0%, HPLC grade) were all purchased from Sigma-Aldrich (USA). High reflectivity mirrors for CRD from Newport optics (USA) around 620 nm with a bandwidth of 583–663 nm, with one-meter radius of curvature, and listed reflectivity greater than 99.9%, were used.

### 2.2. Methods

#### 2.2.1. Gas Injection System

**Figure 3:**
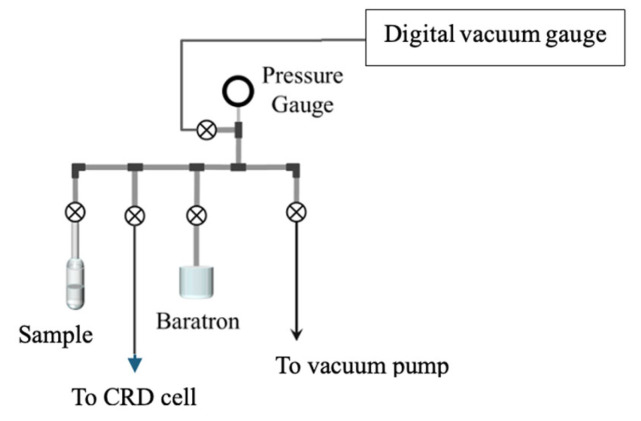
Vacuum and gas injection system.

Figure 3 illustrates the sample introduction system that consists of valves, pressure gauges, and 1/4” stainless steel tubing. The system allows for a vacuum to be applied to the cell for evacuation and sample introduction by using a glass reservoir that contains the liquid sample. The vapor pressure of the sample is the maximum pressure of gas injected into the cell. A digital vacuum gauge measures the vacuum pressure (10^−6^ Torr). The vacuum pressure of the system is achieved with a combination of mechanical and diffusion pumps. A capacitance manometer (Baratron) can measure the absolute pressure of the sample in the cell up to 1000 Torr. A three-way valve allows for venting of the system when required and isolation valves can be used to stop flow in the system. A stainless steel 1/8” tube attaches to the cell via a welded swagelock fitting on the cell. This allows for the introduction of a gaseous sample or a vacuum to be applied to the cell.

#### 2.2.2. Optical Cavity Cell

**Figure 4:**
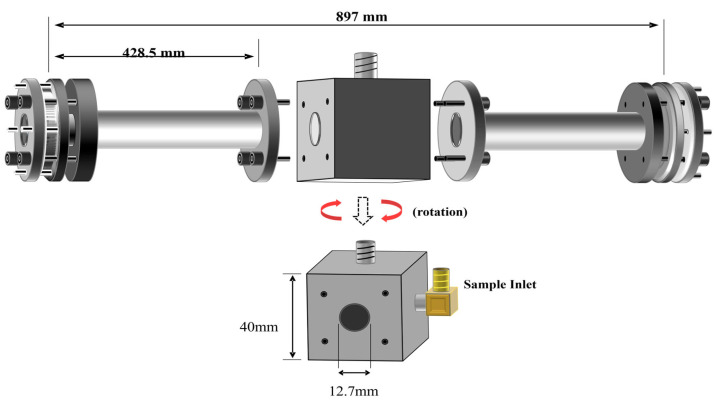
Schematic side view (**top**) of the CRD cell design, (**bottom**) of the center cubic core.

Figure 4 shows the sample cell separated into its several components, as well as the respective dimensions. The sample cell is composed of a central cubic cell with two attaching arms. The central cubic cell was made of yellow brass, whereas the arms are made of aluminum.

The cubic core of this cell measures 40 × 40 × 40 mm^3^ and it serves as the main point of attachment of the cell with the rest of the CRD instrument. The aluminum arms are 428.5 mm in length and 12.7 mm in internal diameter. These arms are attached to the central cubic core with screw head bolts and O-ring seals. The resulting cell is 897 mm in length and 12.7 mm in diameter. Stainless steel tubing is used to connect the cell to the external gas sample preparation system.

**Figure 5:**
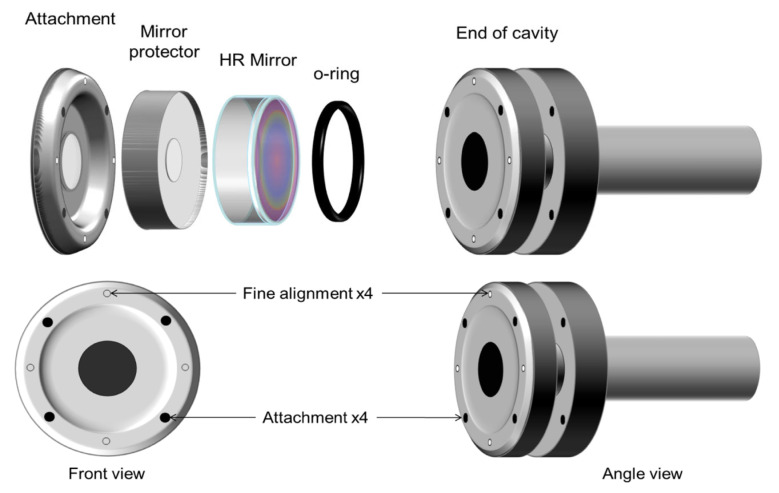
Expanded view of the left arm, showing the O-ring, HR mirror, the mirror protector, and flange attachment (**top**); screw positions needed to hold and finely align the mirrors (**bottom**).

Figure 5 (top) shows a view of the left-hand side of the arm. Each arm consists of two attachment flanges constructed from an aluminum solid piece. The opening in the center of each flange is 12.7 mm wide, as well as the internal diameter of the cell. Between the attachment flange and the high reflector (HR) mirror, an aluminum mirror protector is used to prevent the mirror from breaking during the alignment process. The dimensions of this piece depend on the mirror dimensions, and it is precisely cut to accommodate the mirror based on its thickness and diameter. The HR coating on the mirrors must also be protected from pressure and friction; hence, a rubber O-ring seals the cell from the outside and protects the mirror from mechanical damage. This rubber O-ring must be precisely located concentric with the 12.7 mm opening in the cell, and it must be wide enough to ensure that no leaks will be present when the assembly is complete.

A recommended configuration for the attachment and fine alignment screws is shown in Figure 5 (bottom). It is desirable to design the arms and flanges so that the fine alignment screws result in North, South, East, and West positions. The thread of the attachment screws is ¼″-32, whereas that of the alignment screws is ¼″-80. The system is rigid but there is a small margin of flexibility that allows aligning the mirrors with respect to the laser beam by rotating the alignment screws. This CRD cell design with small modifications has been tested in our laboratory since 2001 at 295 K and in low-temperature studies from 77 K to 298 K [10,11]. The mirror alignment is simpler and more stable in comparison to the alignment with bellows. Mirror alignment is maintained independent of internal or external pressure and temperatures below room temperature. The very rigid nature of our designed cell allows us to measure the angle of −45 degrees in our day-to-day operations. Alignment at room temperature was maintained at low temperatures.

#### 2.2.3. Instrumental Setup for PS-CRD Spectroscopy

**Figure 6:**
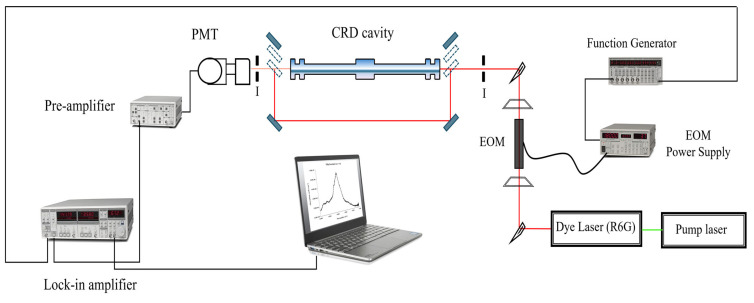
Experimental setup of the PS-CRD spectrometer. Key: EOM, electro-optic modulator; I, iris; L, plane-convex lens; PMT, photomultiplier tube.

The experimental setup is shown in Figure 6. The pump laser is a solid-state Millenia V laser (Spectra Physics), providing a single wavelength (532 nm) at a power of 5W. This laser is used to pump a continuous wave Coherent CR-599 dye laser with a solution of Rhodamine 6G as the fluorescent medium, resulting in a scanning range between 599 and 690 nm or 14,493–16,695 cm^−1^. The wavelength tuning of this dye laser is accomplished with a birefringent filter driven by a stepper motor. This stepper motor is controlled remotely from the computer using LabVIEW^®^. The output beam of the dye laser is modulated into a square wave with a Conoptics model 350-50 electro-optic modulator (EOM). The EOM is driven by a Stanford Research Systems (SRS) function generator, and the modulation frequency depends on the final alignment conditions of the cavity. The beam is injected on-axis into the optical cavity and aligned. The leaked radiation out the back of the cavity is focused by a lens and detected by a Newport 70,680 photomultiplier tube (PMT), which is powered by a high-voltage power supply operated between 700 V and 1000 V.

A combination of movable mirrors and beam splitters is used before and after the cavity to bypass the beam around the cavity and into the PMT. The PMT response is sent to a Stanford Research Systems (SRS) Low-Noise Pre-amplifier model SR 560 for signal amplification and conditioning. The signal is then sent to an SRS 830 Lock-In Amplifier, where the phase angle between the bypassed beam and the leaked beam is determined.

The phase shift (ϕ) is related to the time that the light spends in the cavity or ring-down time (τ) by the equation: $tan\phi \left(\nu \right)=2\pi f\tau$, where *f* is the modulation frequency.

The data from the SRS 830 are sent to a computer via a GPIB interface, where it is also processed using a LabVIEW^®^ code specifically written for this system. The whole system sits on top of a Newport 4′ by 10′ by 8′ optical table, which has vibration isolation legs to eliminate random mechanical noises.

## 3. Results

### Vibrational C-H Fundamental and Overtone Detection

The vibrational overtone spectra of ethyl, ethyl trimethyl, and tert-butyl acetate were obtained for the ∆υ = 1–2 transitions. These spectral regions were measured using a Thermo Nicolet (Nexus 670) Fourier transform spectrophotometer with a resolution of 1 cm^−1^ covering the middle and near-infrared (IR, NIR) regions and a multiple reflection cell with an optical pathlength of 6.6 m at T = 296 K.

**Figure 7:**
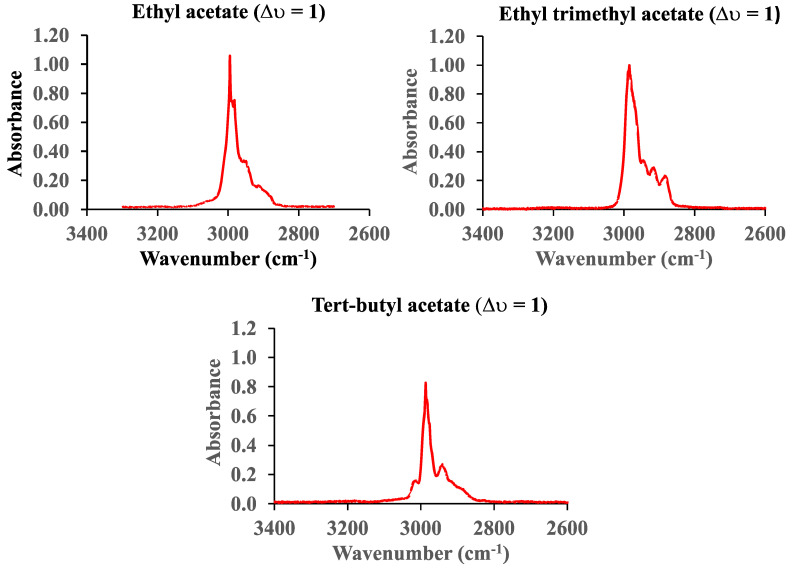
The vibrational fundamental C-H spectra (∆υ = 1) of ethyl, ethyl trimethyl, and tert-butyl acetate.

Figure 7 shows the vibrational fundamental C-H spectrum (∆υ = 1) of ethyl, ethyl trimethyl, and tert-butyl acetate in the range from 3400 cm^−1^ to 2600 cm^−1^. The pressure of ethyl acetate is 0.2 Torr, whereas the pressure of the other two molecules was 0.1 Torr. The pathlength of the cell is 660 cm.

**Figure 8:**
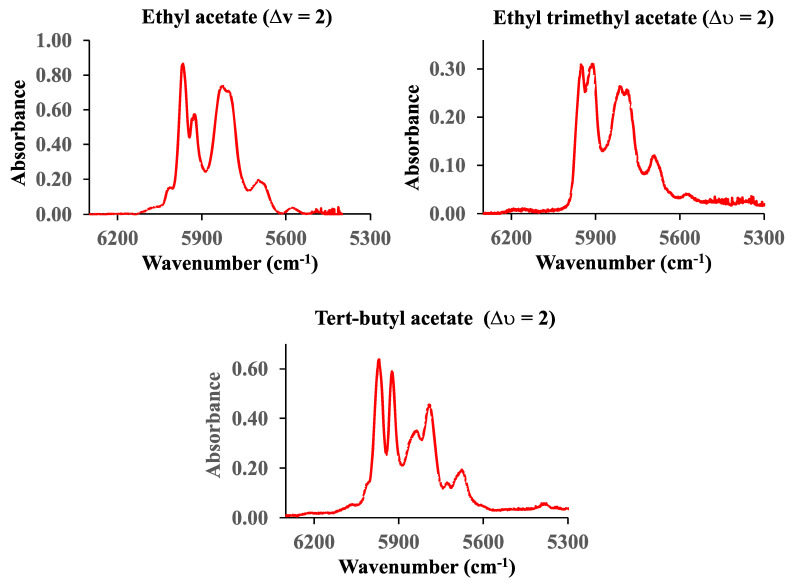
The first (∆υ = 2) C-H vibrational overtone spectra of ethyl, ethyl trimethyl, and tert-butyl acetate.

The vibrational first overtone C-H spectra (∆υ = 2) of ethyl, ethyl trimethyl, and tert-butyl acetate in the range from 6300 cm^−1^ to 5300 cm^−1^ are shown in Figure 8. The pressure of ethyl acetate is 60 Torr, while the pressures of ethyl trimethyl and tert-butyl acetate are 12.3 Torr and 23.6 Torr, respectively. The cell pathlength was 660 cm.

**Figure 9:**
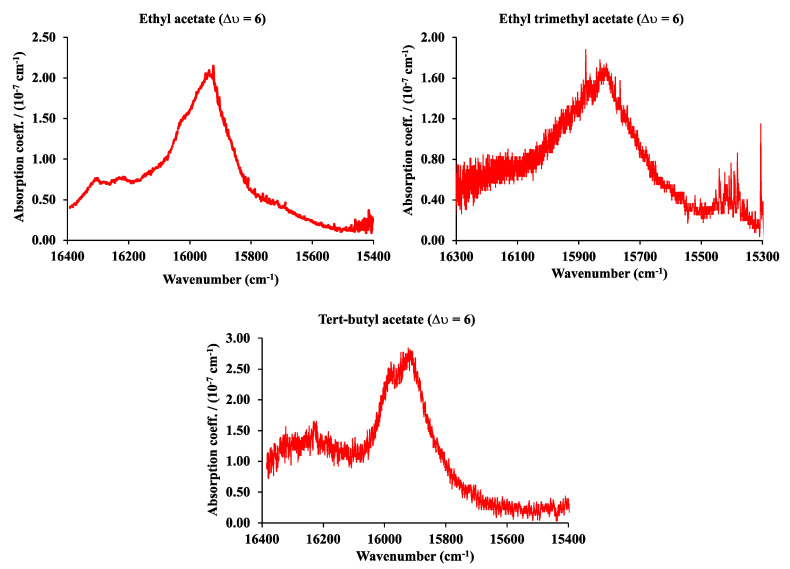
The fifth (∆υ = 6) C-H vibrational overtone spectra of ethyl, ethyl trimethyl, and tert-butyl acetate.

The vibrational fifth (∆υ = 6) overtone C-H spectra of ethyl, ethyl trimethyl, and tert-butyl acetate in the range from 16,400 cm^−1^ to 15,200 cm^−1^ are shown in Figure 9. In this case, the spectra were obtained with the PS-CRD technique described before. The pressure was 60 Torr for ethyl acetate, 12.3 Torr for ethyl trimethyl acetate, and 10 Torr for tert-butyl acetate. The pump laser is a solid-state, frequency-doubled Neodymium Yttrium Vanadium Oxide (Nd: YVO4) laser (Coherent Verdi), providing a single wavelength (532 nm) at a power of 5W. This laser is used to pump a continuous wave (Coherent 899) Ti:Sapphire ring laser with a scanning range between 700 and 800 nm or 12,500–14,300 cm^−1^. The mirrors were provided from Los Gatos, CA, USA, with wavelengths ranging from 740 to 800 nm and 695–745 nm, and a 99.995% reflectivity.

Table 1 summarizes the results at 296 K for peak wavenumber (ν) in cm^−1^, pressure (P) in Torr, molar concentration (c) in mol/L or M, pathlength (l) in cm, absorbance (A), absorption coefficient (a) in cm^−1^, and extinction coefficient (ε) in cm^−1^ M^−1^ of ethyl acetate, ethyl trimethyl acetate, and tert-butyl acetate. It is important to note that, in general, the extinction coefficient (ε) of the fifth overtone (∆υ = 6) is approximately 1 × 10^6^ times smaller than the fundamental transition. The spectra obtained with a conventional FT infrared and near-infrared were obtained with a 660 cm optical pathlength cell, while the PS-CRD has an optical pathlength of 25 km or 2.5 × 10^6^ cm. The measurement of an absorption band around the ∆υ = 6 region indicates that it is possible to measure concentrations as low as 1 × 10^−9^ M in the infrared region between 600 cm^−1^ and 3400 cm^−1^. This concentration corresponds to an absorption coefficient of 1 × 10^−7^ cm^−1^ and a sample pressure of the order of 10^−5^ Torr.

**Figure 10:**
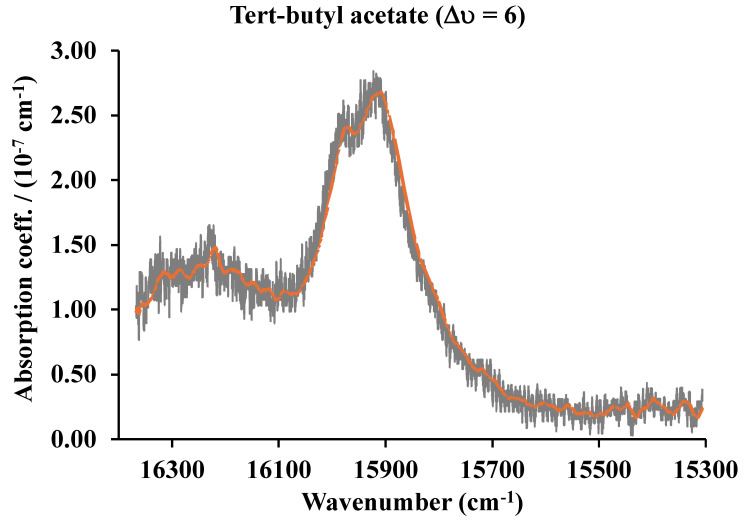
The fifth (∆υ = 6) C-H vibrational overtone spectra of tert-butyl acetate. The averaged signal in red.

With a typical CRD cell in the IR region with an optical pathlength of 10 km, pressures of fractions of mTorr could in principle be detected. As an example, we have measured the second overtone (∆υ = 3) of carbon monoxide around 6350 cm^−1^ [15]. In this case, a near-infrared laser and a 45 cm cavity were used with sample pressures of 10^−3^ Torr at 80 K. As we will show in a later section describing a CRD system in the visible and near-infrared, our projections are very conservative based on results obtained in other laboratories that have determined absorption coefficients for different molecules as low as 10^−12^ cm^−1^ [16,17,18,19]. A noisy signal is very easy to convert to a clean signal with the many algorithms that are in the market. For example, the Microsoft Excel Solver program was used to clean the tert-butyl acetate giving the result shown in Figure 10.

An absorption band of tert-butyl acetate with a peak absorption between 1 and 10% of the band shown could easily be retrieved. This will make the limit of detection around 10^−9^ cm^−1^.

## 4. Discussion

### 4.1. Laboratory and Agricultural Detection of Esters

**Figure 11:**
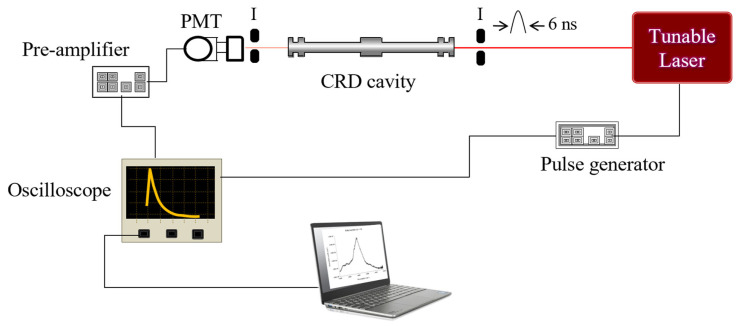
Compact exponential CRD system for vibrational overtone spectra.

A small sample of CRD portable instruments that have been used for measurements of different molecules includes: measurement in the visible region (15,103.91 cm^−1^) of nitrogen oxides NO_3_ and N_2_O_5_ in ambient air [16]; a near-infrared (6046.954, 6049.121 cm^−1^) continuous wave CRD spectrometer for measurements of methane concentrations for planetary surface missions [17]; detection of HO_2_ in the near-IR (6640 cm^−1^) [18]; and breath analysis of acetone for nonintrusive disease diagnosis and metabolic status monitoring [19]. In general, these techniques use high-resolution single-line lasers that produce limits of detection in the range of 10^−10^–10^−12^ cm^−1^. As a prototype, we propose the experimental diagram shown in Figure 11. It is possible to build a compact CRD spectrometer that could be used for measurements in the laboratory using canisters of samples collected in the field. In the future, some modifications will allow for a portable version to be used in the field. For these measurements, high resolution is not required. Small commercial lasers like the Opotek (Opolette 355) tunable laser source (width 18 cm, length 31 cm, height 13 cm) could be used. This laser consists of a pump at 355 nm, and an optical parametric oscillator (OPO) to produce tunable wavelength in the 410–2300 nm range with energy around 9 mJ and a 6 cm^−1^ linewidth. Pulses are 6 ns in duration with up to 20 Hz pulse repetition rate. Wavelength tuning is motorized and computer-controlled. The CRD cell length is 12 cm. We use rigid cells that do not use bellows; thus, permanent alignment is achieved independent of the pressure inside the cell. The light that leaks out the back of the aligned cavity is focused by a lens, detected by a photomultiplier tube powered by a high-voltage power supply, and then sent to a low-noise pre-amplifier connected to a digital oscilloscope. A digital delay/pulse generator initiates the trigger signal to the pump laser and the digital oscilloscope. The CRD signals are measured with the oscilloscope, averaged, and sent to a laptop computer via a GPIB interface, where they are processed by a LabView program written specifically for this system. With this system, vibrational overtones from ∆υ = 2–6 can be recorded. A future portable version of the CRD system, will replace the oscilloscope with an analog-to-digital converter that will send the signals directly to the laptop computer for processing.

### 4.2. Environmental Implications of Ester Reactions

In a study of particle formation in the atmosphere by Zhang et al. [20] and McGraw et al. [21], the following observations have been presented: (1) Atmospheric aerosols contain a high fraction of organic matter. (2) The role of organic compounds is uncertain. (3) Nucleation of sulfuric acid is enhanced by the presence of organic acids. The authors of this paper [21] indicate that the interaction between organic acids and sulfuric acid promotes a very efficient formation of aerosol particles. Benzoic acid has been found to increase the nucleation rate of sulfuric acid by a factor of 5. In addition, larger particle diameters (>10 nm) were detected when benzoic acid concentrations were increased by one or two orders of magnitude. Several mechanisms for the formation of aerosol particles in the atmosphere have been proposed [22]. These particles have an influence on global climate and human health.

Organic acids have vapor pressures that are very small so evaporation from the surface is not the best explanation to account for their presence in the atmosphere. There are many volatile organic compounds that reach the atmosphere. Some researchers indicate the possibility that organic acids are the result of oxidation reactions of organic molecules with radicals in the atmosphere [23]. The role of organic acids in particle formation has been shown to be very important but the origin of organic acids in the atmosphere is not known.

The energy required to excite a molecule to a high vibrational overtone (Δυ = 5, 6, or 7) is in many cases equivalent to the activation energy of unimolecular reactions of some organic molecules. These overtone transitions are induced using visible light. Volatile organic compounds (VOCs) found in the atmosphere originate from anthropogenic and biogenic sources. VOCs may also be formed in the atmosphere as products of reactions of other volatile organic compounds [23,24].

Esters (RCOOR′) decompose at high temperatures and by absorption of UV radiation. It is possible that they could decompose when they are exposed to long periods of visible radiation, forming organic acids, and constituting one of the sources of organic acids in the atmosphere. The reason is that the activation energy necessary to induce their unimolecular decomposition is around 200 kJ/mol. This magnitude of the activation energy is equivalent to the energy of photons of visible light with wavenumbers (or wavelength) around 16,735 cm^−1^ (598 nm). We presume that many organic reactions involving esters take place before they can absorb UV radiation at high altitudes or before they react with radicals (OH, NO_x_, etc). Our explanation for the formation of organic acids in the atmosphere involves the following typical reaction:

Z-COOCH_2_CH_3_ + hν → Z-COOH + CH_2_=CH_2_

In this case, the Z group could be any saturated or aromatic hydrocarbon. In the case of the molecules of this paper, the reactions are:

Ethyl trimethyl acetate: (CH_3_)_3_C-COOCH_2_CH_3_ + hν → (CH_3_)_3_C-COOH + CH_2_=CH_2_
Tert-butyl acetate: CH3-COOC(CH_3_)_3_ + hν → CH3COOH + CH_3_-CH=CH-CH_3_
Ethyl acetate: CH_3_COOCH_2_CH_3_ + hν → CH_3_COOH + CH_2_=CH_2_

In general, esters are not considered harmful to the environment. Typical activation energies obtained from thermal decomposition studies indicate that the magnitudes are around 200 kJ/mol [25,26,27,28]. These reactions have not been studied by one photon decomposition in the visible or ultraviolet. The ∆υ = 6 and 7 C-H vibrational overtone absorption of these molecules is around 16,000 cm^−1^ or 17,000 cm^−1^ (190–200 kJ/mol). This concept related to the origin of some organic acids in the atmosphere is novel and to our knowledge has not been suggested in the literature.

### 4.3. Future Research Directions

This proposal will investigate in the laboratory the possibility of the formation of organic acids in the lower atmosphere from esters absorbing visible light. To this end, we will study the vibrational overtone spectroscopy and potential unimolecular reactions of esters by absorption of visible radiation above 17,000 cm^−1^. Another goal is to construct a portable CRD instrument for the detection of esters in the field.

## Figures and Tables

**Table 1:** Peak wavenumber (ν), pressure (P), molar concentration (c), pathlength (l), absorbance (A), absorption coefficient (α), and extinction coefficient (ε) of ethyl acetate, ethyl trimethyl acetate, and tert-butyl acetate. Temperature 296 K.

Ethyl Acetate							
Transition	ν/cm^−1^	P/Torr	c/M	l/cm	A	α/cm^−1^	ε/cm^−1^M^−1^
Fundamental (∆υ = 1)	2995	0.2	1.1 × 10^−5^	660	1.04	1.6 × 10^−3^	147.6
First overtone (∆υ = 2)	5826	60	3.3 × 10^−3^	660	0.75	1.1 × 10^−3^	3.3 × 10^−1^
Fifth overtone (∆υ = 6)	15,925	60	3.3 × 10^−3^	2.5 × 10^6^	0.21	8.5 × 10^−8^	2.6 × 10^−5^
**Ethyl Trimethyl Acetate**							
**Transition**	**ν/cm^−1^**	**P/Torr**	**c/M**	l **/cm**	**A**	**α/cm^−1^**	**ε/cm^−1^M^−1^**
Fundamental (∆υ = 1)	2985	0.1	5.4 × 10^−6^	660	0.99	1.5 × 10^−3^	277.8
First overtone (∆υ = 2)	5813	12.3	6.7 × 10^−4^	660	0.26	3.9 × 10^−4^	5.8 × 10^−1^
Fifth overtone (∆υ = 6)	15,818	12.3	6.7 × 10^−4^	2.5 × 10^6^	0.43	1.7 × 10^−7^	2.5 × 10^−4^
**Tert-Butyl Acetate**							
**Transition**	**ν/cm^−1^**	**P/Torr**	**c/M**	l **/cm**	**A**	**α/cm^−1^**	**ε/cm^−1^M^−1^**
Fundamental (∆υ = 1)	2987	0.1	5.4 × 10^−6^	660	0.83	1.3 × 10^−3^	240.7
First overtone (∆υ = 2)	5790	23.6	1.3 × 10^−3^	660	0.47	7.1 × 10^−4^	5.5 × 10^−1^
Fifth overtone (∆υ = 6)	15,914	10.0	5.4 × 10^−4^	2.5 × 10^6^	0.70	2.8 × 10^−7^	5.2 × 10^−4^

## Data Availability

Data on this study are available upon request to the corresponding author.
